# Sigma: Strain-level inference of genomes from metagenomic analysis for biosurveillance

**DOI:** 10.1093/bioinformatics/btu641

**Published:** 2014-09-29

**Authors:** Tae-Hyuk Ahn, Juanjuan Chai, Chongle Pan

**Affiliations:** Computer Science and Mathematics Division, Oak Ridge National Laboratory, Oak Ridge, TN, 37831, USA

## Abstract

**Motivation:** Metagenomic sequencing of clinical samples provides a promising technique for direct pathogen detection and characterization in biosurveillance. Taxonomic analysis at the strain level can be used to resolve serotypes of a pathogen in biosurveillance. Sigma was developed for strain-level identification and quantification of pathogens using their reference genomes based on metagenomic analysis.

**Results:** Sigma provides not only accurate strain-level inferences, but also three unique capabilities: (i) Sigma quantifies the statistical uncertainty of its inferences, which includes hypothesis testing of identified genomes and confidence interval estimation of their relative abundances; (ii) Sigma enables strain variant calling by assigning metagenomic reads to their most likely reference genomes; and (iii) Sigma supports parallel computing for fast analysis of large datasets. The algorithm performance was evaluated using simulated mock communities and fecal samples with spike-in pathogen strains.

**Availability and Implementation:** Sigma was implemented in C++ with source codes and binaries freely available at http://sigma.omicsbio.org.

**Contact:** panc@ornl.gov

**Supplementary information:** Supplementary data are available at *Bioinformatics* online.

## 1 INTRODUCTION

It is a public health and national security imperative to prepare for a broad range of biological threats, including infectious diseases and food-borne illnesses (Fox, 2012). Biosurveillance will provide early detection of disease outbreaks and rapid characterization of pathogens. Conventional methods for biosurveillance include laboratory culture, immunoassays, and genotyping (Wagner *et al.*, 2006). Metagenomic sequencing, developed in environmental microbiology, is a promising new method for biosurveillance. For example, fecal samples of suspected patients in food-borne disease outbreaks can be sequenced directly to identify responsible pathogens, bypassing the steps of strain isolation and culturing for pathogen identification. In the 2011 German Shiga toxin-producing *E.coli* (STEC) O104:H4 outbreak, the pathogen was identified by the standard epidemiology procedure of isolation, culturing, and genotyping (Rubino *et al.*, 2011). Multiple isolate cultures of this pathogen from different patients were subsequently sequenced, which generated whole genome sequences for pathophysiology analysis. In the future, metagenomics could be used to sequence fecal samples of patients in the early stage of such an outbreak, potentially allowing more rapid and unambiguous identification of the pathogen.

It is a computational challenge to achieve sensitive and specific identification of pathogens in a complex metagenome background. Many algorithms have been developed to infer the taxonomic composition of a microbial community using metagenomic sequencing data. The algorithms generally used the following three approaches. (i) The *k*-mer statistics approach compares *k*-mer frequency profiles of metagenomic reads with those of organisms representing a wide range of clades. This approach is used by Naïve Bayes Classifier (Rosen *et al.*, 2008), PhymmBL (Brady and Salzberg, 2011), and PhyloPythiaS (Patil *et al.*, 2011). (ii) In the marker gene approach, metagenomic reads are aligned with a set of preselected clade-specific marker genes, and taxonomic classification is inferred from phylogenetic distances to these marker genes. This approach is used by MetaPhlAn (Segata *et al.*, 2012) and AMPHORA2 (Wu and Scott, 2012). Both of the two approaches can produce high-quality classification results at the species level or above without using a significant amount of computation. However, these two approaches may not be adequate for taxonomic classification at the strain level. Genome identification for metagenomic biosurveillance needs to resolve different strains (or serotypes) of the same species. For example, pathogenic *E.coli* strains need to be unambiguously differentiated from coexisting non-pathogenic *E.coli* strains in the metagenome.

Alternatively, (iii) the read mapping approach infers the taxonomic composition of a community by searching metagenomic reads against a database of reference genomes. This approach is used by MEGAN (Huson *et al.*, 2007), Sort-ITEMS (Monzoorul Haque *et al.*, 2009), MetaPhyler (Liu *et al.*, 2011), Grammy (Xia *et al.*, 2011), GASiC (Lindner and Renard, 2013), and Pathoscope (Francis *et al.*, 2013). This approach can provide accurate high-resolution taxonomic classification for clades well represented by reference genomes. It is suitable for metagenomic biosurveillance of known pathogens, which generally have reference genomes available for a variety of strains. However, it is difficult for this approach to detect novel pathogens. When a new strain is present in a sample, its closely related strains in a reference genome database need to be identified and their sequence variations be determined.

Here, we present the Sigma algorithm (strain-level inference of genomes from metagenomic analysis) for metagenomic biosurveillance. A novel probabilistic model was developed to identify and quantify genomes using the read mapping approach. Our benchmarks showed that Sigma provided more accurate strain-level and species-level classification than an established read mapping algorithm—MEGAN, a representative marker gene algorithm—MetaPhlAn, and a recent pathogen detection algorithm—Pathoscope. Sigma was scaled from desktops to supercomputers to achieve a short turnaround time for large metagenomic datasets. This allows a prompt response of a metagenomic biosurveillance network to disease outbreaks using supercomputers.

The existing taxonomic analysis algorithms generally do not provide statistical confidence assessment of their results. It is difficult to make a biosurveillance decision based on the inference of an algorithm on a pathogen’s presence without knowing the uncertainty on such inferences or to use the estimated relative abundance of a genome without knowing the confidence intervals of such estimates. After the identification and quantification of genomes, Sigma can rigorously evaluate the statistical confidence of its findings. Hypothesis testing based on the likelihood ratio test was used to calculate *P*-values for the detection of relevant genomes. Bootstrapping was used to estimate the confidence intervals of the relative abundances of identified genomes.

When an outbreak spreads or a pathogen re-emerges, it is important to determine the sequence variations between the genomes of this pathogen in the field-collected samples and its reference genomes in the database. This allows accurate reconstruction of the transmission sources of the outbreak and the evolutionary history of the pathogen. Thus, biosurveillance further requires information on the single-nucleotide polymorphisms (SNPs) of a detected pathogen to distinguish different variant populations. Identification of variants is straightforward for isolate genome sequencing by mapping all reads to a reference genome. However, in metagenome sequencing, one cannot simply map all metagenomic reads to a reference genome, because some of the aligned reads may originate from different microorganisms, which could introduce false variations. Sigma solves this problem by assigning each read to its most likely originating genome based on the Sigma probabilistic model, which allows subsequent variant calling by variants calling software such as SAMTools (Li *et al.*, 2009).

## 2 ALGORITHM AND IMPLEMENTATION

### 2.1 General description of the Sigma algorithm

**Fig. 1.**
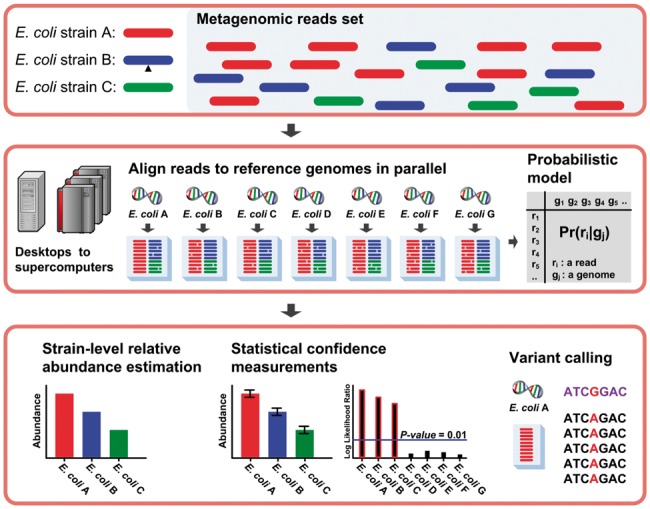
Conceptual overview of the Sigma algorithm. The inputs are metagenomic reads and user-defined reference genomes (top panel). The alignment of reads to genomes is used to define a probabilistic model of metagenomic sequencing (middle panel). Genomes are detected with hypothesis testing, quantified with confidence interval estimation, and scanned for sequence variations (bottom panel).

Figure 1 shows an overview of the Sigma algorithm. Sigma starts with mapping all reads in a metagenomic dataset onto a user-defined database of reference genomes. A parallelization wrapper is provided as an option for users to speedup the read alignment using computer clusters. A read can be aligned to many genomes with up to a given number of mismatches.

The foundation of all statistical inferences in Sigma is a simple probabilistic model for sampling reads with mismatches from genomes at unknown abundances. The Sigma probabilistic model permits maximum likelihood estimation (MLE) of the relative abundances of all genomes measured by the percentages of reads sampled from these genomes. Importantly, we proved the convexity of the objective function of the MLE so that Sigma can guarantee to attain a global optimum solution using interior point non-linear programming (NLP) provided in the Ipopt library (Wachter and Biegler, 2006).

The Sigma probabilistic model further provides the statistical framework for hypothesis testing and confidence interval estimation. Sigma calculates the *P*-value for identification of a genome using a likelihood ratio test of the null hypothesis that this genome is not present in the sample. To account for the quantification uncertainty from the stochastic sampling process of metagenomic sequencing, bootstrapping is used to estimate confidence intervals of relative abundances of identified genomes. As both bootstrapping and likelihood ratio tests entail solving the non-trivial NLP optimization problem for many times, the NLP solver of Sigma is multithreaded and parallelized to computer clusters.

Finally, given the MLE of genome relative abundances, Sigma can calculate the probabilities of sampling a read from its mapped genomes, which allows assigning metagenomic reads to their most likely originating genomes for variant calling. For a selected genome, Sigma provides the alignment of its assigned reads on the genome sequence as the input for variant calling with SAMtools. Sigma filters the variant calling output to identify high-confidence sequence variations.

### 2.2 Mathematical framework of Sigma

The Sigma probabilistic model is used to represent the stochastic process of sampling reads from genomes during metagenomic sequencing. Let $\text{Pr}(g_{j})\in \left[0,1\right]$ be the probability of sampling a random read from the reference genome $g_{j}$. Considering only reads sampled from reference genomes in the database, we have:

$$\sum_{\forall j}\text{Pr}(g_{j})=1.$$

$\text{Pr}(g_{j})$ is determined by the relative abundance, size, and sequencing bias of the genome $g_{j}$. Sigma estimates $\text{Pr}(g_{j})$ from the alignment results using MLE. Consider a read, $r_{i}$, mapped onto $g_{j}$ with z base-pairs of mismatches. A uniform probability of σ (default: 5%) is assumed for any mismatch between a read and a genome, which can stem from genome variability and sequencing errors. Given that a read $r_{i}$ is sampled from $g_{j}$, the probability of obtaining $r_{i}$ with z mismatches and (l−z) matches in the alignment is:

(1) $$\begin{array}{l} \text{Pr}\left(r_{i}|g_{j}\right)=\sigma^{z}{\left(1-\sigma \right)}^{l-z};z\leq U,\\ \text{ Pr}\left(r_{i}|g_{j}\right)=0;z>U, \end{array}$$

where l is the length of $r_{i}$ in base pairs (bp) and U is the maximum number of mismatches allowed in the read alignment. Sigma calculates $\text{Pr}\left(r_{i}|g_{j}\right)$ between each read and each genome based on their alignment, or lack thereof, and populates a constant matrix, Q

$$Q_{i,j}=\text{Pr}\left(r_{i}|g_{j}\right).$$

Then, the probability of sampling $r_{i}$ from $g_{j}$ is simply:

Pr⁡(ri,gj)=Pr⁡(ri|gj)·Pr⁡(gj)=Qi,j·Pr⁡(gj)

Because a read may originate from any one of the reference genomes, the probability of generating $r_{i}$ is:

$$\mathrm{Pr}\left(r_{i}\right)=\underset{\forall j}{\sum }\text{Pr}(r_{i},g_{j}).$$

MLE finds the estimate of $\text{Pr}(g_{j})$ by maximizing the probability of all reads, which is the joint probability of all reads:

$$\max\mathrm{Pr}\left(r_{1},\ldots ,r_{n}\right)$$

$$=\max\prod_{\forall i}\mathrm{Pr}\left(r_{i}\right)$$

$$=\max\prod_{\forall i}\left[\sum_{\forall j}\mathrm{Pr}\left(r_{i},g_{j}\right)\right]$$

$$=\max\prod_{\forall i}\left[\sum_{\forall j}Q_{i,j}\cdot \text{Pr}\left(g_{j}\right)\right].$$

After log-transformation and linear scaling of Q for the convenience of precise numeric calculation, we formulate an optimization problem:
(2)
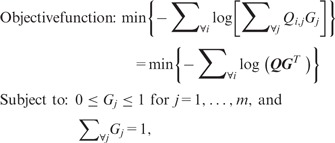

where $G=\left[G_{1},\ldots ,G_{m}\right]=\left[\mathrm{Pr}\left(g_{1}\right),\ldots ,\mathrm{Pr}\left(g_{m}\right)\right]$ for m genomes. This optimization problem is solved using a NLP method (Nocedal and Wright, 2006). Sigma uses the primal-dual interior point NLP method implemented in the Ipopt library (Wachter and Biegler, 2006). The interior point method guarantees global optimum solutions for convex objective functions. The convexity of Sigma’s objective function is proved in Supplementary Proposition 1. Therefore, Sigma can guarantee the maximum likelihood estimates of G. Only aligned reads were considered in the estimation of G. A relative abundance of a genome can be calculated by

(3) $$A\left(g_{j}\right)=G_{j}\cdot \theta ,$$

where θ is the proportion of aligned reads out of all sequenced reads.

Sigma calculates the percentile bootstrap confidence intervals for G. All reads are randomly sampled with replacement to generate a bootstrap resample dataset with the same number of reads as the original dataset. A total of *B* resamples (default: 1000) are generated and Sigma calculates MLEs from the bootstrap resamples: $G_{1}^{*},\ldots ,G_{B}^{*}$, where $G_{k}^{*}=\left[G_{1,k},\ldots ,G_{m,k}\right]$. Let $T_{j}$ be the quantile function for all bootstrap estimates of $G_{j}$, i.e. $\left[G_{j,1},\ldots ,G_{j,B}\right].$ The (1−α) confidence interval of $G_{j}$ is calculated as:

$$(T_{j}\left(\alpha /2\right),T_{j}\left(1-\alpha /2\right)).$$

The bootstrap confidence interval estimation measures the uncertainty caused by the stochastic sampling process of metagenomic sequencing. The confidence interval of $G_{j}$ is converted to the confidence interval of its relative abundance, $A\left(g_{j}\right)$, using Equation (3).

Reference genomes with relative abundances above a certain threshold (default: 0.01%) can be considered to be identified in the metagenome sample. Sigma performs hypothesis testing on the null hypothesis that $g_{j}$ is not present:

$$H_{0}:G=\left[G_{1}\in \left[0,1\right],\ldots ,G_{j}=0,\ldots ,G_{m}\in [0,1]\right],$$

$$H_{1}:G=\left[G_{1}\in \left[0,1\right],\ldots ,G_{j}\in (0,1],\ldots ,G_{m}\in [0,1]\right].$$

The likelihood ratio test is used for the hypothesis testing:

$$\Lambda =\frac{\max\mathrm{Pr}\left(r_{1},\ldots ,r_{n}|H_{0}\right)}{\max\mathrm{Pr}\left(r_{1},\ldots ,r_{n}|H_{1}\right)}.$$

The likelihood ratio compares the maximum likelihood of the data under the null hypothesis versus that under the alternative hypothesis. The likelihood ratio is small if the data strongly supports alternative hypothesis. The likelihood ratio test calculates a *P*-value by assuming a *χ*^2^ distribution with one degree of freedom for the statistic, −2ln⁡Λ. The statistical significance of an identified genome is determined not only by its $G_{j}$, but also by the distinctness of the genome in the database.

### 2.3 Alignment of sequencing reads to reference genomes

The inputs for Sigma are a reference genome database and metagenomic sequencing reads. Sigma can scale up to large databases with tens of thousands of reference genomes. Sigma uses a hierarchical structure for the reference genome database as set up by RefSeq (Pruitt *et al.*, 2012): a root directory containing many sub-directories of FASTA files. Each sub-directory corresponds to a genome and the directory names are used by Sigma as the genome names because they are unique identifiers. A genome may have multiple FASTA files in its sub-directory. Genomes can be added or removed from the reference genome database by simply adding or removing their sub-directories in the root directory. The root directory is specified in the configuration file of Sigma.

Sigma uses a third-party short-read alignment algorithm to align all metagenomic reads onto every reference genome. By default, Bowtie 2 (Langmead and Salzberg, 2012) is used, but users can change to any other short-read alignment algorithm that uses FASTQ/FASTA formats for read input and SAM/BAM formats for alignment output. Users may specify the maximum number of mismatches per read and the range of the inter-mate distance for the read alignment in the Sigma configuration file. The default maximum number of mismatches per read was three for 100-bp reads to balance between the alignment stringency and the computing time of read alignment.

Sigma provides a C++ hybrid parallelization wrapper to scale up the read alignment from multi-core workstations to distributed-memory supercomputers. Computation on a workstation is distributed to multiple processes, each containing 8–24 threads, across CPU cores with shared memory. The Message-Passing Interface (MPI) library is used to distribute the computation across nodes in distributed-memory systems from small clusters to supercomputers. Dynamic load balancing is performed at both the multi-process level and the MPI level to maximize throughput and minimize wall-clock computing time.

### 2.4 Implementation of MLE, bootstrapping, likelihood ratio tests, and variant calling.

Sigma parses the alignment results and generates the *Q* matrix. The relative abundances of genomes are estimated from the *Q* matrix using MLE. Sigma solves the NLP problem for MLE using the Ipopt library (Wachter and Biegler, 2006). Most of the computing time for NLP is spent in the objective function evaluation step, which involves non-trivial linear algebra calculation on the large *Q* matrix. To speedup the calculation, we parallelized the objective functional evaluation step using multi-threading with the Open Multi-Processing (OpenMP) library (Pacheco, 2011).

To perform bootstrapping, Sigma generates a bootstrap *Q* matrix by randomly taking rows from the original *Q* matrix with replacement. Sigma then performs MLE using the bootstrap *Q* matrix. This process is repeated many times to generate a set of bootstrap estimates, which are used to calculate a percentile confidence interval and relative standard deviation (RSD). Sigma distributes the parallel processing of bootstrap samples on a cluster using MPI/OpenMP. Users can set the number of bootstrapping iterations in the Sigma configuration file.

Sigma performs likelihood ratio tests on user-selected genomes. The log-likelihood of the null hypothesis (i.e. a selected target genome is absent) is calculated using a *Q* matrix that does not contain the target genome. The log-likelihood of the alternative hypothesis (i.e. the target genome is present) is the same as calculated from the original *Q* matrix. The log-likelihood of the null hypothesis should be smaller than that of the alternative hypothesis, because the target genome has been estimated to have a non-zero relative abundance. Sigma can perform likelihood ratio tests for many target genomes in parallel on a cluster using MPI/OpenMP.

Sigma assigns metagenomic reads to their most likely reference genomes for variant calling. For each aligned read, the probability of each read from each genome is calculated by:

$$\mathrm{Pr}\left(r_{i},g_{j}\right)=Q_{i,j}\cdot \mathrm{Pr}\left(g_{j}\right).$$

Each read is then assigned to the genome with the highest probability. Sigma generates a BAM alignment file for a selected reference genome using only its assigned reads. SAMtools is then used to identify sequence variants from the BAM file and report results in a variant call format (VCF) file. Finally, the VCF file is processed by Sigma to extract high-confidence sequence variants based on SAMtool’s variant-calling quality score (default: >20) and sequence variant frequency (default: >25%).

All modules of Sigma were implemented in C++. The results are presented in two types of formats: a HTML format for result visualization and a text format for further data analysis.

## 3 SYSTEM AND METHODS

MetaSim (Richter *et al.*, 2008) was used to simulate 100-bp Illumina sequencing reads with an empirical error model. The Illumina error model provided by MetaSim for 80-bp reads was extended to simulate 100-bp reads. The simulated reads contained 0.88 errors per read on average. The preprocessed metagenomic sequencing dataset of a fecal sample from a healthy subject was downloaded from ftp://public-ftp.hmpdacc.org/Illumina/stool/SRS011529.tar.bz2. The Illumina sequencing dataset of the *E.coli* O104:H4 strain 280 (UK Health Protection Agency’s materials identifier H112160280) was downloaded from https://github.com/ngscomparison/NGS-Benchtop-Comparison.

MEGAN (Huson *et al.*, 2007; version 4.70) was downloaded from http://ab.inf.uni-tuebingen.de/software/megan/. Metagenomic datasets were aligned to their corresponding reference genome databases using megablast in BLAST (version 2.2.26) with parameters (–m 7 –v 100 –b 100). MEGAN was run using the default parameters for lowest common ancestor (LCA) except the “Min Support” parameter. The “Min Support” parameter is a threshold for the minimum number of reads that must be assigned to a taxon. It was set to 0.01%, the same as in Sigma.

MetaPhlAn (Segata *et al.*, 2012; version 1.7.7) was downloaded from https://bitbucket.org/nsegata/metaphlan/. The alignment was performed using Bowtie 2 with options specified by MetaPhlAn. Default parameters were used for the MetaPhlAn classification analysis.

Pathoscope (Francis *et al.*, 2013; version 1.0.1) was downloaded from http://sourceforge.net/projects/pathoscope/. BLAST was used for read alignment with suggested parameters (–m 8 –v 100 –b 100) by Pathoscope. Pathoscope was run with the default parameters and the results were reported with the 0.01% relative abundance cutoff (–s 0.01).

All benchmarking was performed on a 64-core computer system with 256 GB shared memory. The scalability of Sigma was also tested on an open-science supercomputer, Titan, at Oak Ridge Leadership Computing Facility.

## 4 RESULTS

### 4.1 Comparison of genome identification and quantification performance

Sigma was compared with three existing taxonomic classification algorithms: MEGAN (Huson *et al.*, 2007), Pathoscope (Francis *et al.*, 2013), and MetaPhlAn (Segata *et al.*, 2012). A 5-genome synthetic community was constructed using MetaSim (Richter *et al.*, 2008) to test the taxonomic resolution of their classification (Table 1 and Supplementary Figure 1). Reads simulated from a target genome, *Escherichia coli* O157:H7 Sakai, were mixed with reads from genomes of the same serotype (*E.coli* O157:H7 TW14359), the same species (*E.coli* K12), the same genus (*E.fergusonii*), and the same family (*Salmonella enterica* serovar Paratyphi A strain ATCC 9150). The average nucleotide identity (ANI; Goris *et al.*, 2007) of the target genome (*E.coli* O157:H7 Sakai) was 99.95% to *E.coli* O157:H7 TW14359, 98.35% to *E.coli* K12, 92.88% to the *E.fergusonii* genome, and 84.47% to the *S.enterica* genome.

**Table 1.** Comparison of Sigma, Pathoscope, MetaPhlAn, and MEGAN in strain-level identification and quantification using a 5-genome synthetic community. (RA%: relative abundance in percentage)

Simulation Input		Sigma		Pathoscope		MEGAN		MetaPhlAn	
Strains	RA(%)	Strains	RA(%)	Strains	RA(%)	Strains/Species	RA(%)	Species	RA(%)
S.enterica serovar Paratyphi A strain ATCC 9150	60	S.enterica serovar Paratyphi A strain ATCC 9150	59.85	S.enterica serovar Paratyphi A strain ATCC 9150	60.11	*S.enterica* serovar Paratyphi A	1.58	*S.enterica*	31.05
						*S.enterica* subsp. enterica	45.49		
						*S.enterica* others	7.78		

E.fergusonii ATCC 35469	27	E.fergusonii ATCC 35469	26.94	E.fergusonii ATCC 35469	27.37	E.fergusonii ATCC 35469	17.49	*E.fergusonii*	28.84

E.coli K12 substr MG1655	9	E.coli K12 substr MG1655	8.91	E.coli K12 substr MG1655	9.64	E.coli K-12	0.02	*E.coli*	13.36

E.coli O157:H7 TW14359	3	E.coli O157:H7 TW14359	3.11	E.coli O157:H7 TW14359	2.6	*E.coli* others	2.19	

E.coli O157:H7 Sakai	1	E.coli O157:H7 Sakai	0.94	E.coli O157:H7 Sakai	0.018	*E.coli* O157:H7	0.02	

						*Enterobacteriacease* others	25.26	*Salmonella* others	26.76

A total of 20 million simulated reads were aligned to all 2266 RefSeq genomes (retrieved in May 2013). The reads from the three *E.coli* strains were mapped to 58 other *E.coli* strains in the database (Supplementary Figure 1a), which challenged the four algorithms’ ability to identify the target *E.coli* O157:H7 Sakai genome and quantify its relative abundance. The relative abundance of the target genome was estimated at 0.94% by Sigma and 0.018% by Pathoscope (Table 1 and Supplementary Figure 1b). The target genome was aggregated with other genomes to the serotype level (*E.coli* O157:H7 at 0.02%) by MEGAN and to the species level (*E.coli* at 13.36%) by MetaPhlAn. Thus, Sigma provided the most accurate quantification for the target *E.coli* genome. However, all algorithms performed relatively well for other genomes at the species level.

Sigma was then compared with the other three algorithms using a more complex synthetic community composed of 100 diverse bacterial and archaeal genomes with relative abundances ranging from 0.41% to 1.59%. The synthetic community covered 35 classes, including 22 genomes from Gammaproteobacteria, 9 from Clostridia, 7 from Betaproteobacteria, and 7 from Bacilli. There were seven *E.coli* strains, two *S.enterica* strains, two *Methanococcus maripaludis* strains, and two *Shewanella baltica* strains. 100 million reads were simulated by MetaSim and the RefSeq database was provided to Sigma, Pathoscope, and MEGAN for read alignment. Supplementary Figure 2 shows the expected relative abundances of all included genomes and the quantification results from the four algorithms. Sigma correctly identified all 100 genomes and accurately quantified their relative abundances within 0.95–1.05 of their expected relative abundances. For the other algorithms, Table 2 shows the numbers of false-positive and false-negative identifications and the numbers of accurate and inaccurate abundance estimations for the correctly identified genomes. Pathoscope was unable to accurately resolve many closely related genomes, such as between the Gammaproteobacteria genomes. MEGAN incorrectly assigned many reads to taxa at higher taxonomic ranks. MetaPhlAn also made many errors at the species level. Overall, this test indicated the superior accuracy of Sigma quantification at the strain and species levels in a complex synthetic community.

**Table 2.** Summary statistics of the identification and quantification results of a 100-genome synthetic community

		Sigma	Patho scope	MEGAN	Meta PhlAn^a^
Expected Genomes/Species		100	100	100	91
True Positive	Accurate RA^b^	100	73	23	6
	Inaccurate RA^b^	0	25	67	71
False Positive		0	0	1	18
False Negative		0	2	10	14

^a^MetaPhlAn performed species-level analysis. Statistics in this column are numbers of species. ^b^True positive identifications may have either accurate RA estimations within 0.95 to 1.05 of their expected RAs or inaccurate RA estimations outside this range.

Biosurveillance requires short turnaround time in data analysis for quick response to outbreaks. The wall-clock analysis time and peak memory usage of the four algorithms were compared based on the 5-genome synthetic community benchmarks on a workstation with 64 CPU cores and 256 GB memory (Table 3). The entire analysis of MetaPhlAn was completed within 10 min using 1 GB memory. Conversely, MEGAN, Pathoscope, and Sigma all required computing-intensive read alignment using BLAST or Bowtie. The abundance estimation by NLP in Sigma was significantly faster and used less memory than the estimation by expectation-maximization (EM) in Pathoscope and by LCA in MEGAN (Table 3). To further speedup the alignment step for large metagenomic datasets, Sigma provides a hybrid parallelization wrapper with dynamic load balancing, which scaled nearly linearly up to 10 000 cores on the supercomputer, Titan. This enabled Sigma to complete the alignment step in only 10 min using 10 000 cores.

**Table 3.** Comparison of analysis time and peak memory usage

	Alignment	Abundance Estimation		Total
	Wall-Clock Time (hr)	Wall-Clock Time (hr)	Memory (GB)	Wall-Clock Time (hr)
Sigma	18	1	62	19
Pathoscope	70	13	118	83
MEGAN	70	12	93	82
MetaPhlAn	N/A	0.2	1	0.2

### 4.2 Statistical confidence assessment by Sigma.

The unique capability of Sigma for statistical confidence assessment was tested using a fecal metagenome dataset (SRS011529) generated by the Human Microbiome Project (HMP; Huttenhower *et al.*, 2012; Methé *et al.*, 2012). To determine the taxonomic composition of the fecal sample, ∼125 million Illumina reads in this dataset was mapped to 24 994 reference genomes from GenBank (retrieved in July 2014). Sigma identified 135 genomes, including many Bacteroidales genomes such as *Prevotella copri, P.stercorea, Bacteroides vulgatus, and B.xylanisolvens* (Supplementary Figure 3). This test also demonstrated the scalability of Sigma to large reference databases.

No *Salmonella* genome was found in this fecal sample. To test detection of *Salmonella* poisoning, varying amounts of reads simulated from a *S.enterica* genome (serovar Paratyphi A strain ATCC 9150) were spiked into the fecal dataset at the relative abundances of 1%, 0.1%, 0.01%, 0.001%, and 0.0001%, which corresponded to genome coverage depths ranging from 27× to 0.0027×. The spike-in fecal metagenomes were aligned with the RefSeq database. There were 26 other genomes from different *S.enterica* strains in the RefSeq database, which served as decoys for testing the statistical uncertainty of strain identification.

**Fig. 2.**
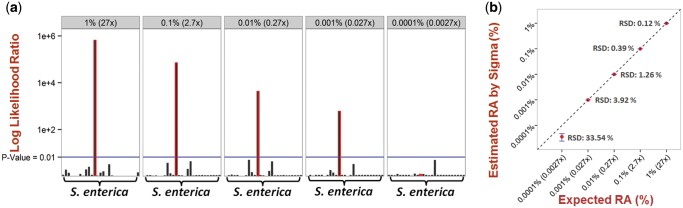
Identification of a *Salmonella* enterica strain at a serial dilution of relative abundances in a human fecal microbiota background. (**a**) Likelihood ratios of all aligned *Salmonella enterica* strains. Only the correct strain (highlighted in red outline) has statistically significant identification with <0.01 *p*-value down to the 0.001% dataset. (b) Estimated and expected relative abundances (RA) of the spike-in *Salmonella enterica* strain. Point estimates (red dots) were bracketed by 95% confidence intervals (blue error bars) with small relative standard deviations (RSD) down to 0.001% (0.027X coverage depth).

Likelihood ratio tests were performed on all *S.enterica* strains in RefSeq in each spike-in dataset (Figure 2a). The correct *S.enterica* strain, and only this strain, was identified with a *P*-value less than 0.01 from 1% down to 0.001% spike-in datasets. As expected, the likelihood ratio of this strain’s identification decreased with its relative abundance. The correct *S.enterica* strain was not identified in the 0.0001% (0.0027×) spike-in dataset. More importantly, using a *P*-value cutoff of 0.01, there was no false-positive identification of other *S.enterica* strains in all spike-in datasets (Figure 2a). We then quantified the spike-in *S.enterica* strain with bootstrap confidence interval estimation (Figure 2b). This strain was quantified at accurate abundances in the 1%–0.001% spike-in datasets, but not in the 0.0001% dataset. The bootstrap confidence intervals were very tight in all except the 0.0001% dataset. The quantification precisions in the five datasets were also measured with RSD of bootstrap estimates. The RSD increased with decreasing amounts of spike-in reads from 1% to 0.0001% (Figure 2b).

To test Sigma’s performance on smaller metagenomic datasets, we repeated the *Salmonella* spike-in experiments using a 10% sub-sample (∼12.5 million reads) and a 1% sub-sample (∼1.25 million reads) of the fecal dataset. Supplementary Figure 4 shows that the target *S.enterica* strain was identified correctly and quantified accurately from all samples with at least 0.027× coverage depth of the target genome, which corresponded to 0.01% spike-in for the 10% sub-sample and 0.1% spike-in for the 1% subsample. Therefore, the detection limit of Sigma was determined by the minimum coverage of a target genome and deeper metagenomic sequencing provided more sensitive detection.

### 4.3 Identification of nearest genomes and detection of strain variations

When a sampled strain is not present in the reference genome database, Sigma can identify its closest relative and detect SNPs between the sampled strain in the metagenome and the identified strain in the database. This capability was tested by searching the above *S.enterica* 1%-spike-in dataset against four different reference genome databases, which represented the spike-in genome as a known strain (Ref1), a novel strain (Ref2), a novel species (Ref3), and a novel genus (Ref4; Supplementary Table 1). When the full RefSeq database (Ref1) was used, Sigma correctly identified and accurately quantified the spike-in strain (serovar Paratyphi A strain ATCC 9150) at 0.988%. Furthermore, Sigma did not find any SNP between the sampled strain and the identified reference strain with 100% genome coverage. This indicated that the identified strain was exactly the same as the sampled strain in the metagenome.

The Ref2 database was constructed by removing the spike-in genome from the RefSeq database. The Ref2 database allowed Sigma to identify a closely related strain of the same serovar, *S.enterica* serovar Paratyphi A strain AKU 12601 (ANI: 99.98%), and quantified its relative abundance at 0.986%. 190 high-confidence SNPs were found on the identified genome of strain AKU 12601 using Ref2, which were verified using the original genome of strain ATCC 9150. 99.95% of the identified genome was covered. The covered region divergence (CRD), defined as the percentage of high-confidence SNPs out of the covered regions of the identified genome, was 0.0042%. The sequence divergence showed a high genome-wide similarity between the identified reference genome and the actually sampled genome.

The Ref3 database was then constructed by removing all *S.enterica* species genomes from the RefSeq database. Sigma identified a genome of the same genus, *S.bongori* NCTC 12419 (ANI: 90.50%), at a relative abundance of 0.031%. The relative abundances and *P*-values calculated by Sigma were based on the covered regions of the identified genomes. Only 11.46% of the identified *S.bongori* genome was covered and 3750 high-confidence SNPs were found on the covered regions with a CRD of 0.734%. This indicated that only a small portion of the identified genomes was related to the sampled genomes in the metagenome.

Finally, the Ref4 database was constructed by removing all *Salmonella* genus genomes from the RefSeq database. Sigma did not identify any genome that was not a background genome in the fecal sample.

### 4.4 Demonstration of Sigma for biosurveillance of the 2011 German *E.coli* outbreak

A hypothetical biosurveillance scenario was constructed based on the 2011 German STEC O104:H4 outbreak (Rubino *et al.*, 2011). The STEC O104:H4 strain 280 (UK Health Protection Agency’s materials identifier H112160280) was isolated from this outbreak and sequenced by Illumina shotgun sequencing (Loman *et al.*, 2012). We spiked 1.7 million reads from this isolate into ∼125 million reads of the fecal metagenomic dataset, which simulated the existence of STEC O104:H4 strain 280 in a fecal sample at a relative abundance of 1.37%. The RefSeq database contained a reference genome, STEC O104:H4 strain 2011C-3493, isolated from a contracted U.S. citizen after travel to Germany during the outbreak (Ahmed *et al.*, 2012). O104:H4 2011C-3493 was closely related to two other O104:H4 genomes in the RefSeq database, 2009EL-2050 and 2009EL-2071, which were isolated from 2009 bloody diarrhea cases in the Republic of Georgia.

After searching the spike-in metagenomic dataset against the RefSeq database, Sigma identified O104:H4 2011C-3493 at 1.14%, O104:H4 2009EL 2050 at 0.18%, and O104:H4 2009EL 2071 at 0.05%. No other genomes were identified from the addition of O104:H4 strain 280. This provided evidence for the presence of the STEC O104:H4 strain, which was very closely related to O104:H4 2011C-3493, and further to its two immediate phylogenetic neighbors. Sigma detected 211 high-confidence SNPs on the O104:H4 2011C-3493 genome from the metagenomic dataset. 99.96% of the O104:H4 2011C-3493 genome was covered with 0.004% CRD.

## 5 DISCUSSION

A number of taxonomic classification algorithms have been developed for metagenomic data with different advantages. For example, the LCA algorithm used by MEGAN can provide classification at a higher taxonomic rank for a novel microorganism without a reference genome from a closely related strain or species in the database. The marker gene approach used by MetaPhlAn only requires a small amount of computing resources for large metagenomic datasets. The general-purpose taxonomic classification algorithms (Brady and Salzberg, 2011; Diaz *et al.*, 2009; Liu *et al.*, 2011; Monzoorul Haque *et al.*, 2009; Patil *et al.*, 2011; Xia *et al.*, 2011) typically provide comprehensive phylogenetic coverage and identifications at proper taxonomic ranks for complex microbial communities, which is important for many microbial ecology studies.

Conversely, Pathoscope and Sigma both are designed for detecting pathogens in metagenomic samples. However, Pathoscope did not provide accurate resolution of different strains in a sample, which is required for biosurveillance to distinguish pathogenic strains from non-pathogenic strains and to pinpoint the serotype of a pathogen from a complex metagenome background. This is probably because the EM algorithm used by Pathoscope can only provide a local optimum solution for genome abundance estimation. The local optimum solution may deviate significantly from the global optimum solution for metagenomes that are complicated by multiple different strains. However, the NLP algorithm used by Sigma mathematically guarantee a global optimum solution for genome abundance estimation, due to the convexity of the objective function for the MLE.

Furthermore, Sigma provides three unique capabilities needed for metagenomic biosurveillance. First, Sigma can evaluate the statistical confidence of genome identification and quantification with hypothesis testing and confidence interval estimation. The uncertainty quantification will support more informed decision making in biosurveillance. Second, Sigma can determine the divergence of the actual genomes in the sample from the reference genomes in the database by variant calling. By assigning metagenomic reads to their most likely originating genomes, Sigma makes the metagenomic SNP analysis amenable to the existing variant calling algorithms designed for isolate genomes. Strain variations can be used to track the divergence of a pathogen during its spread. Finally, Sigma users can optionally use high-performance computing to achieve fast analysis with short turnaround time. The computation is scalable to very large metagenomic datasets.

## Supplementary Material

Supplementary Data
