# Supplementary material for: Sigma: Strain-level inference of genomes from metagenomic analysis for biosurveillance
Source: Bioinformatics. 2014 Sep 29;31(2):170–7. doi: 10.1093/bioinformatics/btu641 (PMC4287953; doi:10.1093/bioinformatics/btu641)
Supplement: Supplementary Data [file supp_31_2_170__index.html]

Sigma: Strain-level inference of genomes from metagenomic analysis for biosurveillance — Sigma: Strain-level inference of genomes from metagenomic analysis for biosurveillance — Supplementary Data 

# Sigma: Strain-level inference of genomes from metagenomic analysis for biosurveillance

## Supplementary Data

files

**Files in this Data Supplement:**

- Supplementary Data - pdf file
- Supplementary Data - pdf file
