# Supplementary material for: Sigma: Strain-level inference of genomes from metagenomic analysis for biosurveillance
Source: Bioinformatics. 2014 Sep 29;31(2):170–7. doi: 10.1093/bioinformatics/btu641 (PMC4287953; doi:10.1093/bioinformatics/btu641)
Supplement: Supplementary Data [file supp_btu641_Sigma_Supplementary.pdf]

Supplementary Figure 1. Detection of an *E. coli* strain from a 5-genome synthetic community. (a) Alignment of reads to many genomes related to the five mixed genomes highlighted in red outline. (b) Accurate estimation of the relative abundances of the five correctly identified genomes by Sigma. The genomes are listed in the same order in the two bar charts.

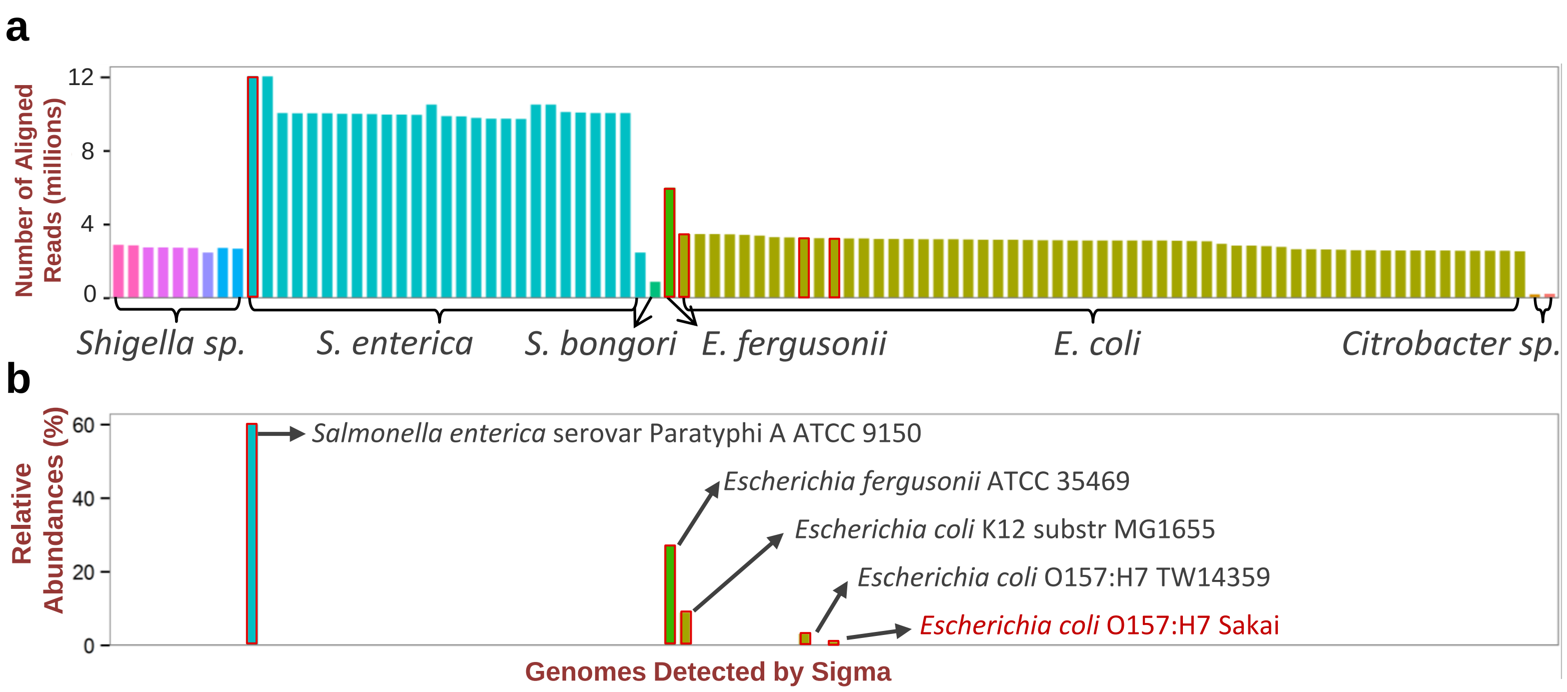

Supplementary Figure 2. Comparison of Sigma with Pathoscope, MetaPhlAn and MEGAN on a 100-genome synthetic community. The quantification accuracy of an algorithm is measured by the ratios of the estimated relative abundances (RA) to the expected RA (i.e. a ratio of 1 for perfect estimation) across the 100 genomes. The genomes are listed alphabetically in the x-axis labeled by their genome or species indices. The legend shows the names and the expected RA of the 100 genomes. Species represented by multiple genomes are highlighted in red in the legend.

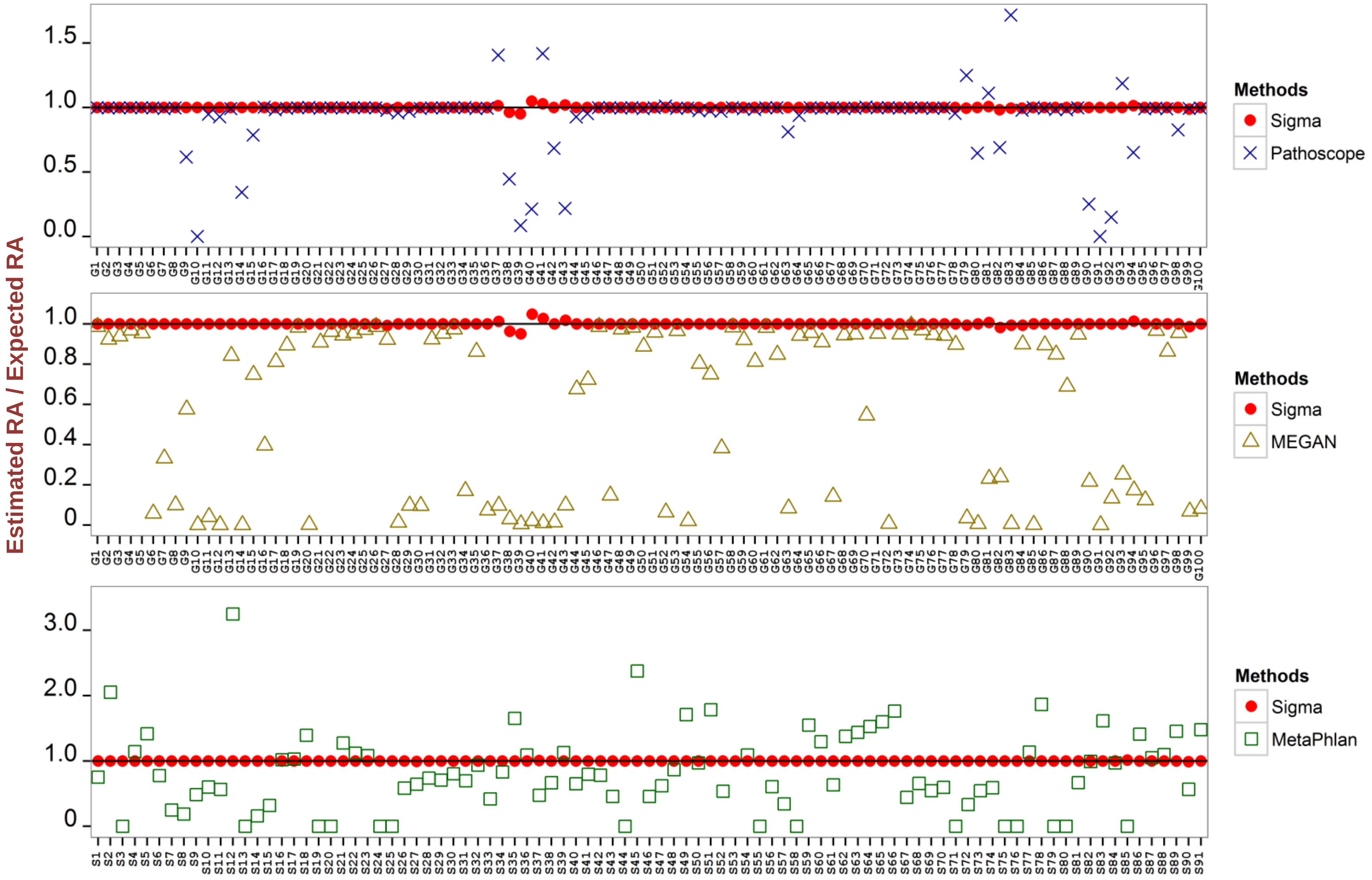

Genome Index (Species Index)	Geome Name	Expected RA	Genome Index (Species Index)	Geome Name	Expected RA	Genome Index (Species Index)	Geome Name	Expected RA
G1(S1)	Acidobacterium capsulatum ATCC 51196	0.52	G35(S35)	Dictyoglomus turgidum DSM 6724	0.51	G69(S62)	Pyrobaculum aerophilum IM2	0.99
G2(S2)	Aciduliprofundum boonei T469	0.59	G36(S36)	Enterococcus faecalis OG1RF	0.99	G70(S63)	Pyrobaculum arsenaticum DSM 13514	0.84
G3(S3)	Aerococcus urinae ACS 120 V Col10a	0.75	G37(S37)	Escherichia coli 042	0.77	G71(S64)	Pyrobaculum calidifontis JCM 11548	0.7
G4(S4)	Akkermansia muciniphila ATCC BAA 835	0.51	G38(S37)	Escherichia coli ATCC 8739	0.54	G72(S65)	Pyrococcus furiosus DSM 3638	0.61
G5(S5)	Archaeoglobus fulgidus DSM 4304	0.96	G39(S37)	Escherichia coli K 12 substr MG1655	0.61	G73(S66)	Pyrococcus horikoshii OT3	0.46
G6(S6)	Bacillus amyloliquefaciens DSM 7	1.33	G40(S37)	Escherichia coli O104 H4 2011C 3493	0.41	G74(S67)	Rhodopirellula baltica SH 1	0.82
G7(S7)	Bacillus cereus ATCC 10987	1.3	G41(S37)	Escherichia coli O55 H7 CB9615	1.09	G75(S68)	Ruegeria pomeroyi DSS 3	1.28
G8(S8)	Bacillus thuringiensis BMB171	1.41	G42(S37)	Escherichia coli O7 K1 CE10	0.66	G76(S69)	Salinispora arenicola CNS 205	0.63
G9(S9)	Bacteroides thetaiotaomicron VPI 5482	1.56	G43(S37)	Escherichia coli UMN026	0.56	G77(S70)	Salinispora tropica CNB 440	1.36
G10(S10)	Bacteroides vulgatus ATCC 8482	1.05	G44(S38)	Escherichia fergusonii ATCC 35469	0.86	G78(S71)	Salmonella bongori NCTC 12419	0.48
G11(S11)	Bordetella bronchiseptica RB50	0.52	G45(S39)	Fusobacterium nucleatum ATCC 25586	1.16	G79(S72)	Salmonella enterica serovar Paratyphi A ATCC 9150	1.56
G12(S12)	Buchnera aphidicola TLW03 Acyrthosiphon pisum	0.75	G46(S40)	Gemmatimonas aurantiaca T 27	0.5	G80(S72)	Salmonella enterica serovar Typhimurium	1.04
G13(S13)	Burkholderia 383	0.52	G47(S41)	Geobacter sulfurreducens PCA	0.48	G81(S73)	Shewanella baltica OS185	1.55
G14(S14)	Burkholderia pseudomallei 668	1.25	G48(S42)	Haloferax volcanii DS2	1.07	G82(S73)	Shewanella baltica OS223	0.56
G15(S15)	Burkholderia xenovorans LB400	1.48	G49(S43)	Herpetosiphon aurantiacus DSM 785	0.84	G83(S74)	Shigella flexneri 2002017	1.59
G16(S16)	Caldicellulosiruptor bescii DSM 6725	1.44	G50(S44)	Hydrogenobaculum Y04AAS1	1.14	G84(S75)	Streptomyces venezuelae ATCC 10712	1.58
G17(S17)	Caldicellulosiruptor saccharolyticus DSM 8903	1.33	G51(S45)	Ignicoccus hospitalis KIN4 I	1.32	G85(S76)	Sulfobacillus acidophilus DSM 10332	1.18
G18(S18)	Calditerrivibrio nitroreducens DSM 19672	0.71	G52(S46)	Klebsiella pneumoniae 1084	1.16	G86(S77)	Sulfolobus tokodaii 7	0.98
G19(S19)	Candidatus Accumulibacter phosphatis clade IIA UW 1	1.4	G53(S47)	Leptothrix cholodnii SP 6	0.49	G87(S78)	Sulfurihydrogenibium azorense Az Fu1	1.51
G20(S20)	Candidatus Tremblaya princeps PCIT	1.13	G54(S48)	Listeria monocytogenes ATCC 19117	1.16	G88(S79)	Sulfurihydrogenibium YO3AOP1	0.69
G21(S21)	Carboxydothermus hydrogenoformans Z 2901	0.86	G55(S49)	Methanocaldococcus jannaschii DSM 2661	1.59	G89(S80)	Thermincola potens JR	1.42
G22(S22)	Chlorobium limicola DSM 245	1.01	G56(S50)	Methanococcus maripaludis C5	1.58	G90(S81)	Thermoanaerobacter pseudethanolicus ATCC 33223	1.21
G23(S23)	Chlorobium phaeobacteroides DSM 266	0.48	G57(S50)	Methanococcus maripaludis S2	1	G91(S82)	Thermobaculum terrenum ATCC BAA 798	1.46
G24(S24)	Chlorobium phaeovibrioides DSM 265	0.42	G58(S51)	Methanopyrus kandleri AV19	0.5	G92(S83)	Thermotoga neapolitana DSM 4359	1.16
G25(S25)	Chlorobium tepidum TLS	1.33	G59(S52)	Methanosarcina acetivorans C2A	1.11	G93(S84)	Thermotoga petrophila RKU 1	1.46
G26(S26)	Chloroflexus aurantiacus J 10 fl	0.54	G60(S53)	Nanoarchaeum equitans Kin4 M	1.51	G94(S85)	Thermotoga RQ2	0.74
G27(S27)	Citrobacter koseri ATCC BAA 895	1.31	G61(S54)	Nitrosomonas europaea ATCC 19718	1.49	G95(S86)	Thermus thermophilus HB8	1.26
G28(S28)	Clostridium botulinum A ATCC 19397	0.82	G62(S55)	Nostoc PCC 7120	0.88	G96(S87)	Thiobacillus denitrificans ATCC 25259	1.39
G29(S29)	Clostridium difficile 630	1.21	G63(S56)	Pantoea ananatis	1.54	G97(S88)	Treponema denticola ATCC 35405	1.34
G30(S30)	Clostridium thermocellum ATCC 27405	1.21	G64(S57)	Pectobacterium carotovorum PC1	1.19	G98(S89)	Wolinella succinogenes DSM 1740	1.22
G31(S31)	Cytophaga hutchinsonii ATCC 33406	0.52	G65(S58)	Pelodictyon phaeoclathratiforme BU 1	0.78	G99(S90)	Yersinia pestis CO92	1.51
G32(S32)	Deinococcus radiodurans R1	1.02	G66(S59)	Persephonella marina EX H1	0.95	G100(S91)	Zymomonas mobilis ZM4	0.89
G33(S33)	Desulfovibrio desulfuricans ATCC 27774	0.53	G67(S60)	Porphyromonas gingivalis ATCC 33277	1.19			
G34(S34)	Desulfovibrio vulgaris DP4	0.64	G68(S61)	Providencia stuartii MRSN 2154	0.49			

Supplementary Figure 3. Taxonomic composition of a fecal metagenome estimated by Sigma using 24,994 reference genomes from GenBank. (a) Names and relative abundances of identified genomes. (b) Pie chart of relative abundances of identified genomes and unaligned reads. (c) Pie chart of percentages of aligned reads assigned to identified genomes.

a

Genome Name	RA (%)	Genome Name	RA (%)	Genome Name	RA (%)	Genome Name	RA (%)
Prevotella copri DSM 18205	9.981	*Parabacteroides* sp. 20_3	0.244	*Parabacteroides* sp. D13	0.059	*Clostridium leptum* DSM 753	0.019
Prevotella stercorea DSM 18206	3.554	*Bacteroides* sp. 3_1_23	0.215	*Roseburia intestinalis* L1-82	0.055	*Bacteroides salyersiae* WAL 10018	0.019
Bacteroides vulgatus ATCC 8482	3.067	*Bacteroides dorei* CL02T12C06	0.208	*Bacteroides xylanisolvens* SD CC 1b	0.054	*Clostridium* sp. JCC	0.018
Bacteroides vulgatus CL09T03C04	1.812	*Bacteroides coprophilus* DSM 18228	0.198	*Alistipes finegoldii* DSM 17242	0.048	*Blautia wexlerae* DSM 19850	0.018
Bacteroides vulgatus dnLKV7	1.454	*Bacteroidales bacterium* ph8	0.196	*Roseburia intestinalis* XB6B4	0.045	*butyrate-producing* bacterium SS3/4	0.018
Bacteroides xylanisolvens XB1A	1.285	*Clostridium* sp. L2-50	0.180	*Bacteroides* sp. 2_1_33B	0.044	*Prevotella bergensis* DSM 17361	0.018
Bacteroides sp. 3_1_40A	1.031	*Butyricimonas virosa* DSM 23226	0.179	*Bacteroides fragilis* str. Korea 419	0.043	*Parabacteroides* sp. D25	0.018
Bacteroides sp. 4_3_47FAA	0.929	*Capnocytophaga* sp. oral taxon 329 str. F0087	0.166	*Roseburia intestinalis* M50/1	0.041	*Bifidobacterium adolescentis* L2-32	0.017
Bacteroides ovatus SD CMC 3f	0.922	*Odoribacter splanchnicus* DSM 220712	0.163	*Faecalibacterium* cf. prausnitzii KLE1255	0.039	*Bacteroides* sp. D2	0.017
Bacteroides uniformis ATCC 8492	0.918	*Bacteroides xylanisolvens* CL03T12C04	0.158	*Alistipes senegalensis* JC50	0.038	*Dorea formicigenerans* 4_6_53AFAA	0.017
Bacteroides sp. 1_1_30	0.862	*Bacteroides* sp. 9_1_42FAA	0.137	*Bacteroides massiliensis* dnLKV3	0.038	*Parabacteroides distasonis* CL03T12C09	0.016
Bacteroides uniformis CL03T12C37	0.790	*Bacteroides xylanisolvens* SD CC 2a	0.125	*Alistipes onderdonkii* DSM 19147	0.037	*Parabacteroides distasonis* ATCC 8503	0.016
Bacteroides vulgatus PC510	0.782	*Eubacterium eligens* ATCC 27750	0.121	*Faecalibacterium prausnitzii* M21/2	0.034	*Catenibacterium mitsuokai* DSM 15897	0.016
Parabacteroides merdae ATCC 43184	0.753	*Bacteroides massiliensis* B84634	0.119	*Bacteroides cellulosilyticus* CL02T12C19	0.033	*Ruminococcus* sp. 5_1_39BFAA	0.015
Parabacteroides merdae CL09T00C40	0.749	*Bacteroides* sp. 3_1_33FAA	0.106	*Bacteroides fragilis* str. 3397 T10	0.033	*Barnesiella intestinihominis* YIT 11860	0.015
Bacteroides sp. 1_1_6	0.735	*Eubacterium ventriosum* ATCC 27560	0.102	*Bacteroides ovatus* 3_8_47FAA	0.033	*Clostridium* sp. M62/1	0.015
Dialister invisus DSM 15470	0.701	*Bacteroides pectinophilus* ATCC 43243	0.098	*Bacteroides massiliensis* B84634	0.032	*Bacteroides cellulosilyticus* DSM 14838	0.014
Bacteroides thetaiotaomicron VPI-5482	0.504	*Bacteroides fragilis* 638R	0.093	*Bacteroides* sp. 3_1_19	0.032	*Prevotella multisaccharivorax* DSM 17128	0.014
Bacteroides stercoris ATCC 43183	0.492	*Eubacterium rectale* DSM 17629	0.091	*Ruminococcaceae bacterium* D16	0.032	*Porphyromonas bennonis* JCM 16335	0.013
Parabacteroides merdae CL03T12C32	0.444	*Paraprevotella xylaniphila* YIT 11841	0.090	*Bacteroides caccae* CL03T12C61	0.031	*Parabacteroides johnsonii* DSM 18315	0.013
Bacteroides sp. D20	0.415	*Bacteroides dorei* DSM 17855	0.090	*Faecalibacterium prausnitzii* SL3/3	0.031	*Bacteroides cellulosilyticus* WH2	0.013
Bacteroides sp. D22	0.397	*Roseburia inulinivorans* DSM 16841	0.081	*Prevotella* sp. BV3P1	0.029	*Bacteroides* sp. 14(A)	0.013
Bacteroides faecis MAJ27	0.395	*Eubacterium rectale* ATCC 33656	0.079	*Ruminococcus callidus* ATCC 27760	0.029	*Sutterella wadsworthensis* 3_1_45B	0.013
Paraprevotella clara YIT 11840	0.372	*Bacteroides dorei* CL03T12C01	0.073	*Faecalibacterium prausnitzii* A2-165	0.029	*Oscillibacter* sp. KLE 1745	0.012
Bacteroides uniformis dnLKV2	0.369	*Bacteroides ovatus* CL02T12C04	0.072	*Ruminococcus torques* L2-14	0.028	*Bacteroides fragilis* str. DS-71	0.012
Bacteroides finegoldii DSM 17565	0.366	*Eubacterium rectale* M104/1	0.071	*Faecalibacterium prausnitzii* L2-6	0.026	*Dorea* sp. AGR2135	0.012
Alistipes putredinis DSM 17216	0.364	*Roseburia hominis* A2-183	0.070	*Coprococcus comes* ATCC 27758	0.024	*Ruminococcus bromii* L2-63	0.012
Bacteroides stercoris CC31F	0.345	*Alistipes indistinctus* YIT 12060	0.068	*Subdoligranulum* sp. 4_3_54A2FAA	0.023	*Prevotella bivia* DSM 20514	0.011
Bacteroides sp. 1_1_14	0.323	*Alistipes shahii* WAL 8301	0.068	*Alistipes* sp. HGB5	0.023	*Prevotella timonensis* 4401737	0.011
Bacteroides sp. 4_1_36	0.287	*Bacteroides nordii* JCM 12987	0.064	*Bacteroides rodentium* JCM 16496	0.022	*Bacteroides fragilis* str. 3783N1-8	0.011
Bacteroides thetaiotaomicron dnLKV9	0.277	*Bacteroides ovatus* CL03T12C18	0.062	*Bacteroides* sp. 2_1_16	0.021	*Collinsella aerofaciens* ATCC 25986	0.011
Bacteroides sp. D1	0.273	*Bacteroides finegoldii* CL09T03C10	0.062	*Dorea longicatena* DSM 13814	0.021	*Candidatus Alistipes* marseilloanorexicus AP11	0.010
Bacteroides dorei 5_1_36/D4	0.267	*Bacteroides ovatus* ATCC 8483	0.061	*Bacteroides uniformis* CL03T00C23	0.020	*Coprococcus catus* GD/7	0.010
Bacteroides sp. 2_2_4	0.259	*Dorea formicigenerans* ATCC 27755	0.059	*Sutterella wadsworthensis* HGA0223	0.019	*Bacteroides coprocola* DSM 17136	0.010

b

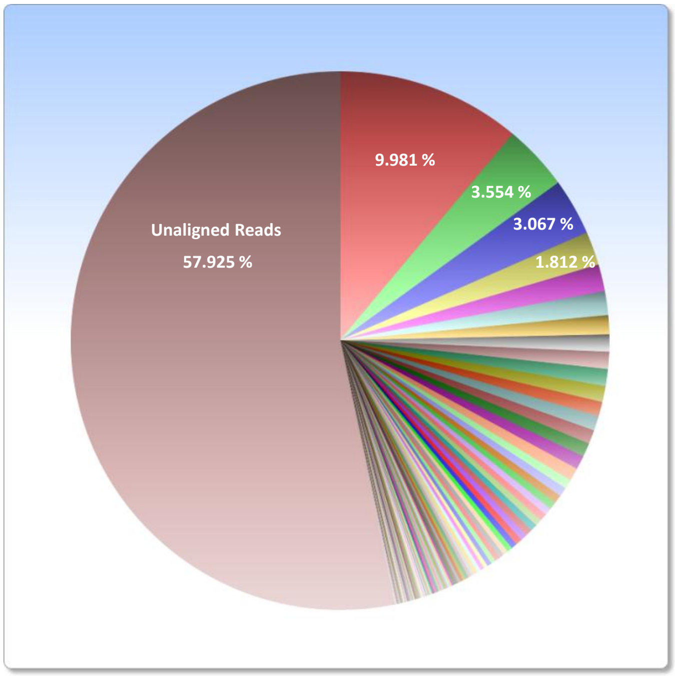

c

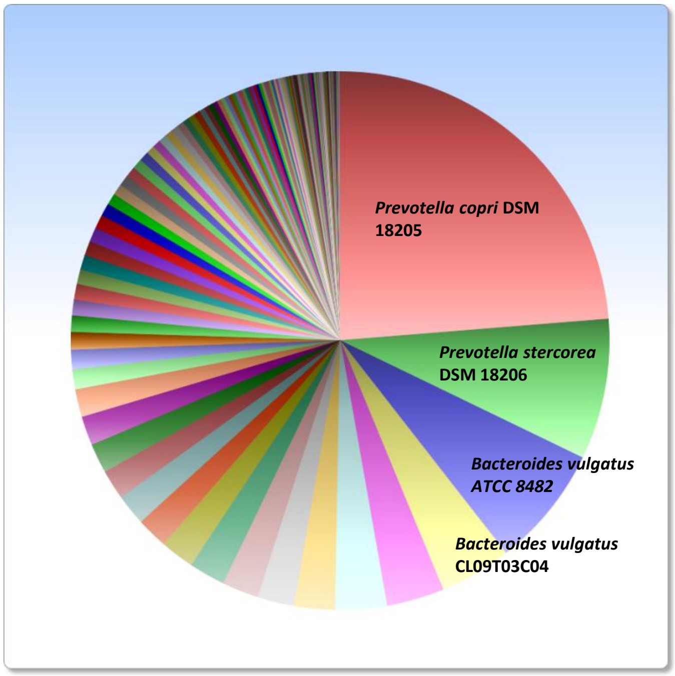

Supplementary Figure 4. Identification of a *Salmonella enterica* strain at a serial dilution of relative abundances in sub-samples of a human fecal metagenome. Various amounts of *Salmonella enterica* reads were spiked into the full dataset [(a) and (b)], a 10% sub-sample [(c) and (d)], and a 1% sub-sample [(e) and (f)] of a human fecal metagenome. (a), (c) and (e) show the likelihood ratios of all aligned *Salmonella enterica* strains in these samples. (b), (d), and (e) show the estimated and expected relative abundances (RA) of the spike-in *Salmonella enterica* strain in these samples.

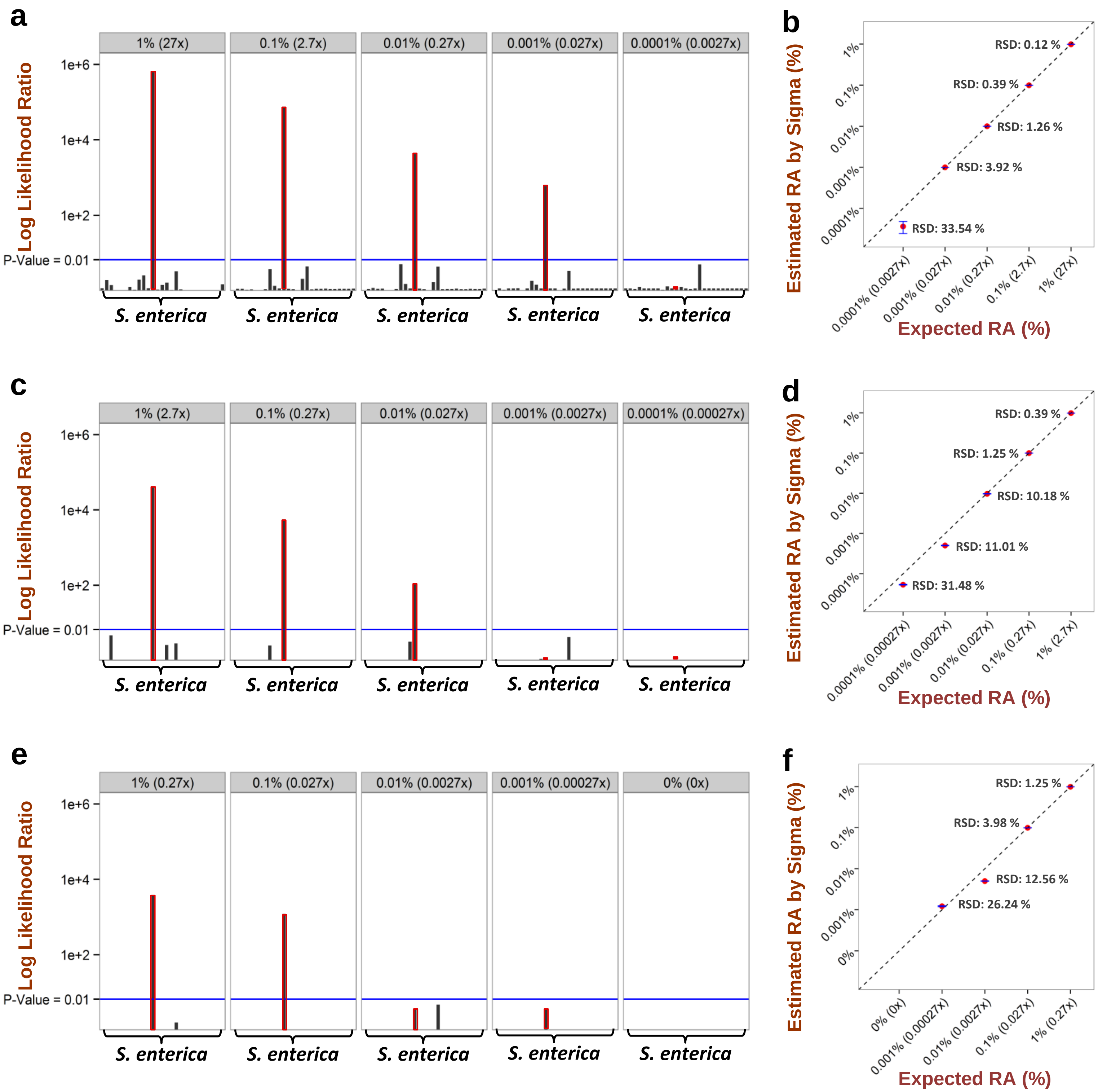

Supplementary Table 1. Identification of a *S. enterica* strain in a human fecal microbiota background using different reference genome databases.

Test	Identified Genome	Relative Abundance (%)	†ANI % (SD %)	Genome Coverage (%)	High-confidence SNPs	‡Covered Region Divergence (%)
[1]Ref1	*Salmonella enterica* serovar Paratyphi A ATCC 9150	0.988		100	0	0
[2]Ref2	*Salmonella enterica* serovar Paratyphi A AKU 12601	0.986	99.99 (0.08)	99.95	190	0.004
[3]Ref3	*Salmonella bongori* NCTC 12419	0.031	90.50 (3.39)	11.46	3750	0.734
[4]Ref4	None					

† ANI % (SD %): average nucleotide identity and its standard deviation in percentage.

‡ Coverage region divergence: the percentage of high-confidence SNPs out of the covered regions of the identified genome.

[1] Ref1 (2266 genomes): the full RefSeq database

[2] Ref2 (2265 genomes): the RefSeq database without the spike-in genome from *Salmonella enterica* serovar Paratyphi A ATCC 9150

[3] Ref3 (2239 genomes): the RefSeq database without all *Salmonella enterica* species genomes

[4] Ref4 (2238 genomes): the RefSeq database without all *Salmonella* genus genomes
