# Supplementary material for: Sigma: Strain-level inference of genomes from metagenomic analysis for biosurveillance
Source: Bioinformatics. 2014 Sep 29;31(2):170–7. doi: 10.1093/bioinformatics/btu641 (PMC4287953; doi:10.1093/bioinformatics/btu641)
Supplement: Supplementary Data [file supp_btu641_Sigma_SupplementaryProposition.pdf]

Supplementary Proposition 1

Proof for the convexity of the MLE objective function

PROPOSITION 1:

The MLE objective function

$$f(\boldsymbol{x}) = -\sum_{i=1}^{n} \log\left[\sum_{j=1}^{m} Q_{i,j} x_j\right]$$

is convex for all $\boldsymbol{x} = [x_1, \cdots, x_m]$ *and* $x_j \in [0,1]$ *where* $0 \leq Q_{i,j} \leq 1$.

Proof:

By definition, a function $f : \mathbb{R}^n \to \mathbb{R}$ is convex if and only if for any two different points $\boldsymbol{x}_{(1)}$ and $\boldsymbol{x}_{(2)}$ in the domain of f and any real number λ such that $0 \leq \lambda \leq 1$, one has

$$f\left[\lambda \boldsymbol{x}_{(1)} + (1-\lambda)\boldsymbol{x}_{(2)}\right] \leq \lambda f\left(\boldsymbol{x}_{(1)}\right) + (1-\lambda) f\left(\boldsymbol{x}_{(2)}\right). \qquad (1)$$

Since summation of convex functions is convex, it suffices to prove that $g(\boldsymbol{x}) = -\log\left[\sum_{j=1}^{m} Q_{i,j} x_j\right] = -\log(\boldsymbol{q_i} \cdot \boldsymbol{x})$ is convex for every i where $\boldsymbol{q_i} = \left[Q_{i,1}, \cdots, Q_{i,m}\right]$. Now the left hand side of Equation (1) applying to $g(\boldsymbol{x})$ is

$$g\left[\lambda \boldsymbol{x}_{(1)} + (1-\lambda)\boldsymbol{x}_{(2)}\right] = -\log\left[\boldsymbol{q_i} \cdot \left(\lambda \boldsymbol{x}_{(1)} + (1-\lambda)\boldsymbol{x}_{(2)}\right)\right]$$
$$= -\log\left[\left(\lambda\, \boldsymbol{q_i} \cdot \boldsymbol{x}_{(1)} + (1-\lambda)\, \boldsymbol{q} \cdot \boldsymbol{x}_{(2)}\right)\right].$$

And the right hand side of (1) is

$$\lambda g\left(\boldsymbol{x}_{(1)}\right) + (1-\lambda) g\left(\boldsymbol{x}_{(2)}\right) = -\left[\lambda \log(\boldsymbol{q_i} \cdot \boldsymbol{x}_{(1)}) + (1-\lambda)\log(\boldsymbol{q_i} \cdot \boldsymbol{x}_{(2)})\right].$$

Since $\log(x)$ is a concave function of x, obviously $g(\boldsymbol{x})$ is a convex function of $\boldsymbol{x}$. Therefore, the objective function $f(\boldsymbol{x})$ is convex.
